# Target Engagement of Lecanemab on CSF Aβ Protofibril Toxic Species in Clarity AD

**DOI:** 10.1002/alz70861_108918

**Published:** 2025-12-23

**Authors:** Kanta Horie, Akihiko Koyama, Pallavi Sachdev, Kazuyoshi Shuta, Kazuhiro Tahara, Michio Kanekiyo, Shobha Dhadda, Larisa Reyderman, Steven Hersch, Michael C. Irizarry, Lynn D Kramer

**Affiliations:** ^1^ Eisai Inc., Nutley, NJ USA; ^2^ Eisai Co., Ltd., Tsukuba, Ibaraki Japan

## Abstract

**Background:**

Soluble amyloid‐β protofibrils (Aβ‐PF) are synaptotoxic species and are in equilibrium with amyloid plaques. Lecanemab is a humanized IgG1 monoclonal antibody that binds with high affinity to Aβ protofibrils. We developed a sensitive immunoassay using lecanemab as the capture antibody that selectively quantifies Aβ‐PF in cerebrospinal fluid (CSF), and found CSF Aβ‐PF levels significantly increased in Alzheimer’s disease (AD) and correlated with neurodegeneration biomarkers (Shinohara‐Noguchi, Ann Neurol. 2025). In this report, we further characterized CSF Aβ‐PF in the Phase 3 study of lecanemab (Clarity AD): (1) at baseline, (2) longitudinally in placebo arm (natural history), and (3) demonstrated the target engagement (*in vivo* protofibril binding) of lecanemab.

**Method:**

CSF samples collected from the Clarity AD were analyzed using the developed immunoassay. The assay is tolerant to therapeutic lecanemab in the CSF, and measures total Aβ‐PF (bound and unbound). 410 subjects (Placebo=207, Lecanemab=203) had a CSF sample at baseline and 253 subjects (Placebo=127, Lecanemab=126) had a CSF sample at baseline and at least one post‐baseline sample. Other biomarker assessments including amyloid‐PET, and CSF biomarkers including neurogranin were described previously and used for correlation analyses.

**Result:**

Baseline CSF Aβ‐PF showed significant correlation with neurogranin, a biomarker of synaptic dysfunction (r=0.153, *p* =0.0127). In the placebo arm, there was an increase from baseline in CSF Aβ‐PF levels which significantly correlated with the increase from baseline observed in CSF neurogranin levels. In the lecanemab‐treatment arm, the CSF total Aβ‐PF levels (free and bound) increased relative to placebo at each of the post‐baseline assessments (*p* =0.0126 at 12 months and numerically higher at 18 month). In the subjects achieving amyloid‐negativity defined as Centiloid<30 at 18 months, lecanemab‐treatment group demonstrated a larger percent change from baseline compared with subjects who remained amyloid‐positive (Centiloid≥30) at 18 months.

**Conclusion:**

The novel immunoassay for the CSF Aβ‐PF indicates that soluble Aβ‐PF is quantifiable in human CSF *in vivo*. Increase in total (free and bound) CSF Aβ‐PF with lecanemab‐treatment is consistent with binding of Aβ‐PF to lecanemab (target engagement), and mobilization of Aβ‐PF from amyloid plaques (pharmacodynamic effect), confirming the unique dual mechanism of lecanemab targeting toxic Aβ‐PF and plaques.

